# Positive experience with TNF-α inhibitor in toxic epidermal necrolysis resistant to high-dose systemic corticosteroids

**DOI:** 10.3389/fmed.2023.1210026

**Published:** 2023-07-24

**Authors:** Ekaterina A. Nikitina, Daria S. Fomina, Ulyana A. Markina, Sergey S. Andreev, Yuri V. Streltsov, Tatiana S. Kruglova, Marina S. Lebedkina, Alexander V. Karaulov, Maryana A. Lysenko

**Affiliations:** ^1^City Clinical Hospital No. 52, Moscow, Russia; ^2^The First Sechenov Moscow State Medical University (Sechenov University), Moscow, Russia; ^3^Department of General Therapy, Pirogov Russian National Research Medical University, Moscow, Russia

**Keywords:** toxic epidermal necrolysis (Stevens-Johnson syndrome/toxic epidermal necrolysis), toxic epidermal necrolysis, TNF-α inhibitor, TNF-α inhibitor/anti TNF-α, Stevens-Johnson syndrome, Etancercept

## Abstract

Stevens-Johnson syndrome (SJS) and toxic epidermal necrolysis (TEN) are rare, potentially life-threatening syndromes characterized by the development of necrotic epidermal and mucosal lesions. The most common etiologic cause of SJS/TEN is drug-induced mechanisms. The group of drugs with high potential risk includes sulfonamides, anticonvulsants, non-steroidal anti-inflammatory drugs (NSAIDs), allopurinol, phenobarbital, etc. There is no gold standard treatment algorithm for SJS/TEN. In medical practice, systemic glucocorticosteroids (sGCS), intravenous immunoglobulin (IVIG), plasmapheresis, and cyclosporine are used empirically and in various combinations. Recently published studies have demonstrated the efficacy of TNF-α inhibitors as a promising approach in SJS/TEN, including cases resistant to high-dose sGCS, with etanercept and infliximab being the most commonly used drugs. In a large multicenter study by Zhang J et al. (XXXX), 242 patients treated with etanercept, sGCS, or a combination of both had lower mortality compared to the control group. A shorter skin healing time was documented compared to sGCS monotherapy, thus reducing the risk of secondary infections. The published data show a high efficacy with THF-α inhibitor blockade, but the safety of TNF-α inhibitors in patients with SJS/TEN is still questionable due to the paucity of available information. As all clinical research data should be accumulated to provide reliable evidence that the use of TNF-α inhibitors may be beneficial in SJS/TEN, we report a case of etoricoxib-associated SJS with progression to TEN in a 50-year-old woman who was refractory to high-dose sGCS therapy.

## Introduction

Stevens-Johnson syndrome (SJS) and toxic epidermal necrolysis (TEN) are rare, potentially life-threatening syndromes, characterized by the development of necrotic lesions of the epidermis and mucous membranes. The study by Seminario-Vidal L. et al. showed that the incidence of SJS/TEN ranges from 1.58 to 2.26 cases per 1 million people in the USA, with a mortality rate of 15 to 49% (1).

According to the classification, SJS and TEN are one disease and differ in severity, prognosis, and percentage of skin and mucosal involvement; alternatively, SJS and TEN are considered different stages of the same disease (2).

Drug-induced mechanisms are the most common etiologic cause of SJS/TEN. High-risk drugs include sulfonamides, anticonvulsants, non-steroidal anti-inflammatory drugs (NSAIDs), allopurinol, phenobarbital, etc. (3). There is no gold standard treatment algorithm for SJS/TEN. In clinical practice, systemic glucocorticosteroids (sGCS) (4, 5), intravenous immunoglobulin (IVIG) (3), plasmapheresis (6), and cyclosporine (7) are used empirically and in combination.

Secondary infection is a serious clinical complication in SJS/TEN with an incidence of 85%, with sepsis being the most common cause of death. Fatal complications include acute renal failure, liver dysfunction, acute respiratory distress syndrome, and thromboembolic complications (8).

Previous studies have reported an increased risk of infectious complications and a higher mortality rate in patients treated with sGCS (5, 9–11). However, a study by Kardaun SH et al. showed that the short-term use of high-dose sGCS in the early stages of SJS/TEN reduces mortality and is not associated with infectious complications (5).

IVIG is one of the most commonly used treatment options with controversial evidence-based data. According to the European Study on Severe Skin Adverse Reactions (EuroSCAR), IVIG monotherapy does not reduce mortality compared to maintenance therapy (3). In a cohort study of 28 cases, no differences in mortality were found between high-dose (≥3 g/kg) and low-dose (< 3 g/kg) regimens (12).

The mechanism of plasmapheresis is to remove causative triggers for the development of SJS/TEN, as well as chemokines and cytokines involved in the pathogenesis of this disease. Narita YM et al. reported that plasmapheresis is effective in TEN patients resistant to sGCS therapy (13). However, another study concluded that plasmapheresis as monotherapy is ineffective and does not affect overall survival in patients with SJS/TEN (14).

The immunosuppressive mechanism of cyclosporine, which inhibits the cytotoxic effect of T lymphocytes, FasL, nuclear factor-kB, and tumor necrosis factor-alpha (TNF-α), may be an option (15). Limited case reports and meta-analyses have shown that cyclosporine treatment reduces mortality in patients with SJS/TEN (16–19).

Recently published studies have demonstrated the efficacy of TNF-α inhibitors as a promising approach in SJS/TEN, including cases resistant to high-dose sGCS (20, 21), with etanercept and infliximab being the most commonly used drugs (8, 20–27). In a large multicenter study by Zhang et al. 242 patients treated with etanercept, sGCS, or a combination of both had lower mortality compared to the control group. A shorter skin healing time was documented compared to sGCS monotherapy, thus reducing the risk of secondary infections (27). The published data show a high efficacy with THF-α inhibitor blockade, but the safety of TNF-α inhibitors in patients with SJS/TEN is still questionable due to the paucity of available information. As all clinical research data should be accumulated to provide reliable evidence that the use of TNF-α inhibitors may be beneficial in SJS/c, we report a case of etoricoxib-associated SJS with progression to TEN in a 50-year-old woman who was refractory to high-dose sGCS therapy.

## Objectives

This study aims to highlight the unmet need in SJS/TEN treatment algorithms and to present a successful case of treatment with TNF-α inhibitor-etanercept in a patient unresponsive to high-dose sGCS therapy.

## Case description

Patient P, a 50-year-old woman, was admitted to the hospital 5 days after the onset of symptoms with a primary diagnosis of Stevens-Johnson syndrome.

According to the patient's history, she had been taking etoricoxib 60 mg/day for 10 days as an analgesic before noticing a sore throat, painful swallowing, and a papular rash in the neck area. The next day, the patient discontinued the treatment on her own. On day 3, the rash spread to the upper extremities (forearms and hands), painful erosions appeared in the oral cavity, and the body temperature increased to 38.0°C. Prior to hospitalization, dexamethasone 8 mg and cetirizine 10 mg were administered once without response. The patient denied other concomitant diseases and medications. On the day of admission, the patient presented with a subfebrile temperature (37.8°C) and macular-papular lesions with a tendency to coalesce involving the chest, back area, and upper and lower extremities. Isolated skin bullae were observed on the feet, hands, and chest; oral involvement included mucosal hyperemia, multiple erosions, and angular cheilitis. There were also features of conjunctivitis and genital tract mucosal membrane involvement. Initially, Nicolsky's sign was negative, the lesion area was < 10%, and the diagnosis was Stevens-Johnson syndrome. Laboratory results showed elevated levels of the systemic inflammatory response (SIR) markers: fibrinogen was up to 6.95 g/l (2.76–4.71), ferritin was up to 125 μg/l (20–120), CRP was up to 29.42 mg/l (0.00–6.00), LDH was up to 261.6 UD/l (0.0–248.0), urine analysis with leukocyturia (75.0 cl/mL), and bacteriuria (420.0 cl/mL). PCR of the oropharyngeal swabs for SARS-CoV-2 RNA was negative. SCORTEN on admission was 1 (probability of hospital death 4%) (age over 40 years) (28).

According to the algorithm for determining the culprit drug (ALDEN) (29), etoricoxib was considered a likely factor in the development of SJS.

Treatment was initiated with intravenous methylprednisolone 325 mg/day, followed by topical chlorhexidine and dexpanthenol solutions. Benzildimethyl [3-(myristoylamino)propyl]ammonium chloride monohydrate and dexamethasone (0.1%) were added as recommended by the ophthalmologist. Antibacterial treatment included amoxicillin+sulbactam 1.5 g three times daily intravenously for suspected lower urinary tract infection based on the patient's complaints and conditions of painful urination, subfebrile temperature, elevated systemic inflammatory response (SIR) markers, leukocyturia, and bacteriuria.

**Figure 1:**
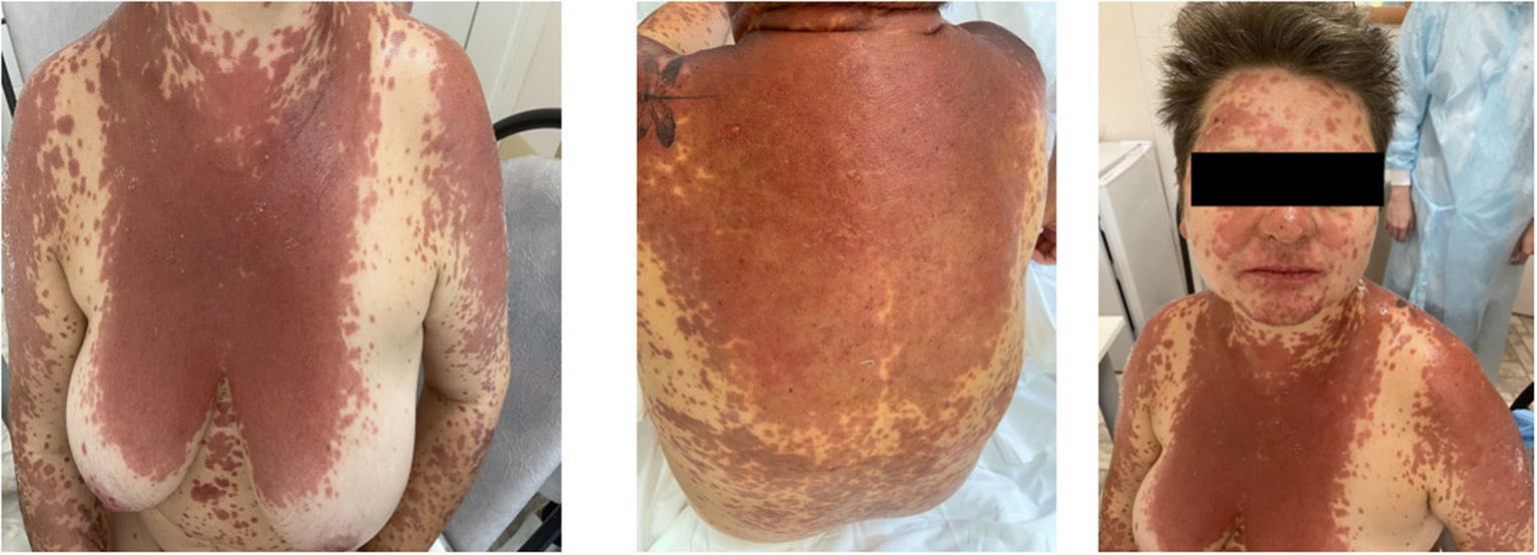
Progressing skin lesions on day 5 of hospitalization.

Evaluation of the treatment response showed the normalization of body temperature and regression of SIR markers (CRP and fibrinogen were within reference values). However, despite the sGCS treatment, the progression of the pathological process was observed; the area of the affected skin surface expanded with new erosions on the mucous membranes, so on day 3 of hospitalization, the dose of methylprednisolone was increased to 500 mg/day. The subsequent rash spread reached more than 30%, involving the face, chest, upper and lower extremities, with erosions on the back, and legs. Bullae filled with serous contents appeared on the palmar and plantar surfaces, chest, and back. The spectrum of new complaints included nasal pain and hemorrhagic crusts on the red border of the lips (Figure 1). Nikolsky's sign proved to be positive.

A repeat SCORTEN assessment was performed with a score of 2 (age over 40 years, epidermal detachment >10%), meeting the criteria for toxic epidermal necrolysis. The phase of sGCS resistance was reached, and the interdisciplinary medical committee decided to start the therapy with TNF-a inhibitor etanercept 50 mg after obtaining the patient's informed consent. The dose of sGCS was de-escalated due to the high risk of side effects (secondary infections, thrombosis, and skin and mucosal healing disorders). The cumulative dose of sGCS on day 6 was 2,900 mg. After initiation of etanercept, the dynamic clinical and laboratory monitoring showed no additional adverse events registration: no fever, CRP, and fibrinogen remained within reference values (Table 1).

**Table 1:** Dynamics of laboratory parameters.

	**Day 1**	**Day 5**	**Day 11**
CRP (mg/l)	29.42	2.36	0.92
Fibrinogen (g/l)	6.95	2.67	2.85
ALT (UD/l)	37.5	49.0	38.1
AST (UD/l)	33.4	20.3	20.8
Serum albumin (g/l)	44.1	36.3	35.4
Serum creatinine (μmol/l)	90.5	84.4	88.4
Leukocyte (x10^*^9/l)	8.6	9.6	8.6

**Figure 2:**
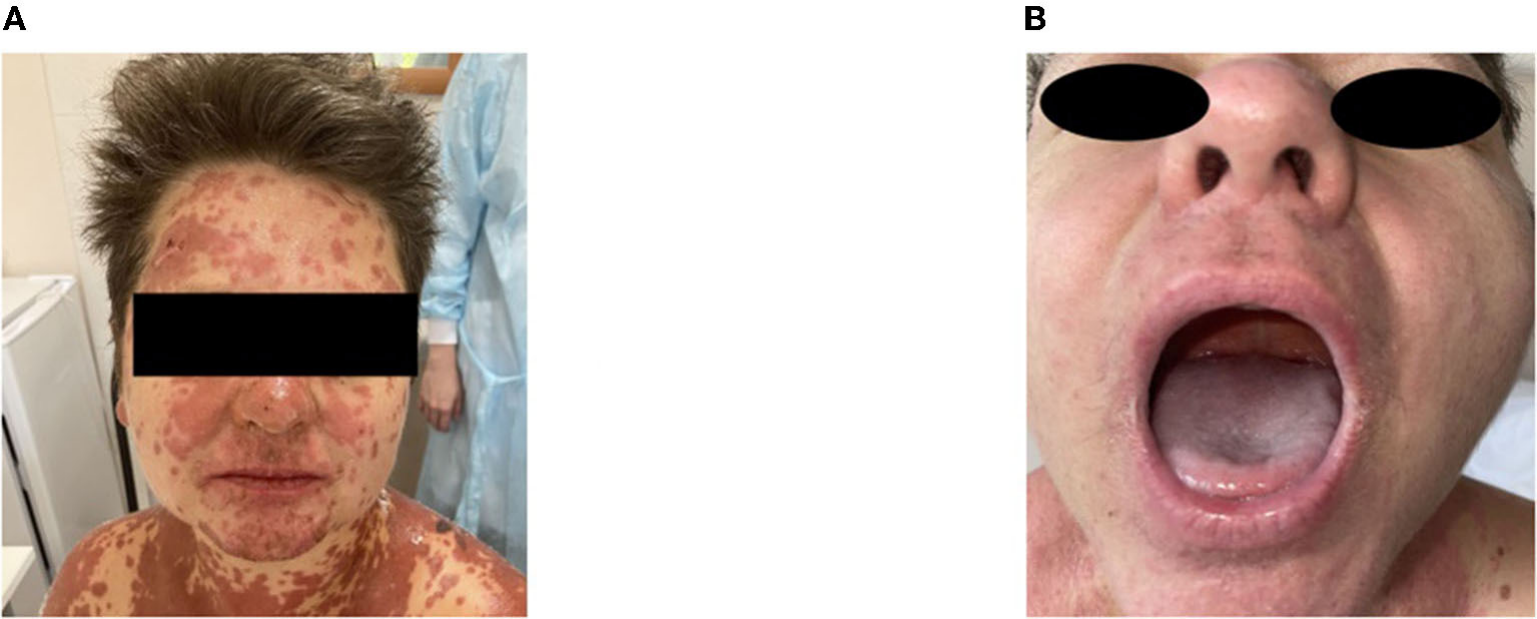
Skin process dynamics. **(A)** On the day of etanercept administration, palmar surfaces featured multiple bullae filled with serous content followed by erosive defects. **(B)** On day 11, previously erosive surfaces were epithelialized, and residual desquamation and depigmentation were present in the previously affected areas.

**Figure 3:**
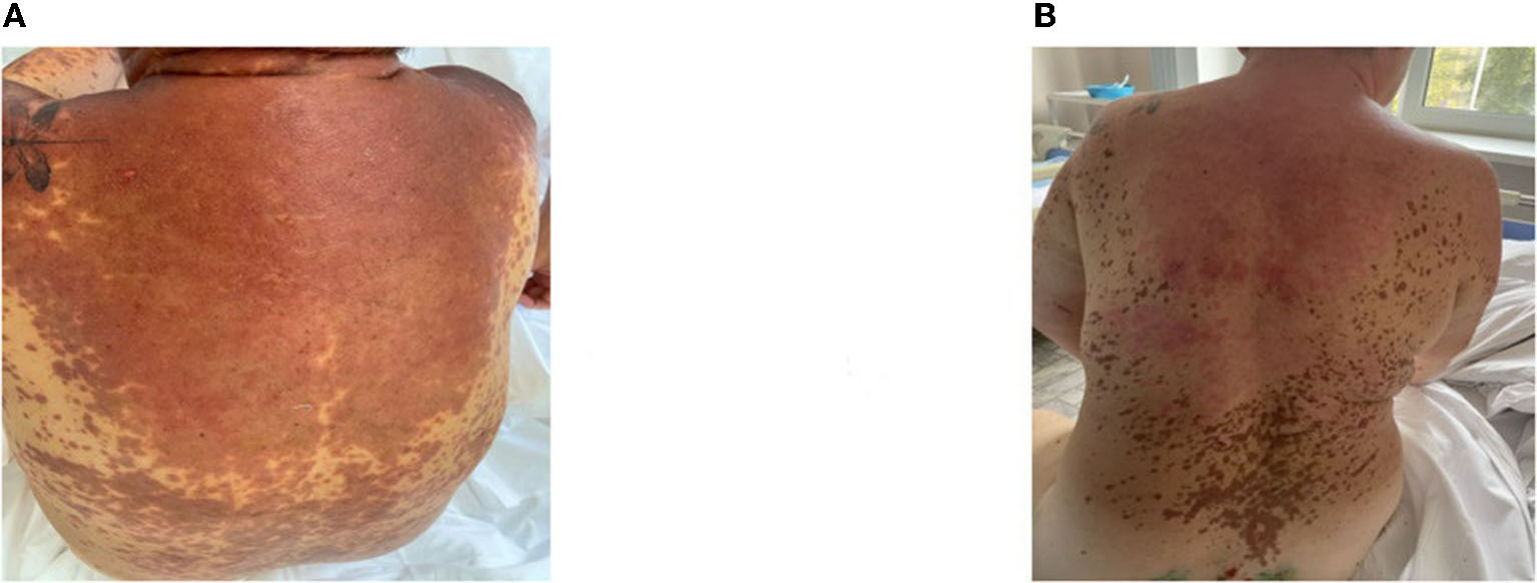
Skin process dynamics. **(A)** On the day of etanercept administration, multiple erosive areas with confluent erythematous rashes on the back were observed. **(B)** On day 11, we observed epithelialized previously erosive areas and foci of depigmentation in previously affected areas.

**Figure 4:**
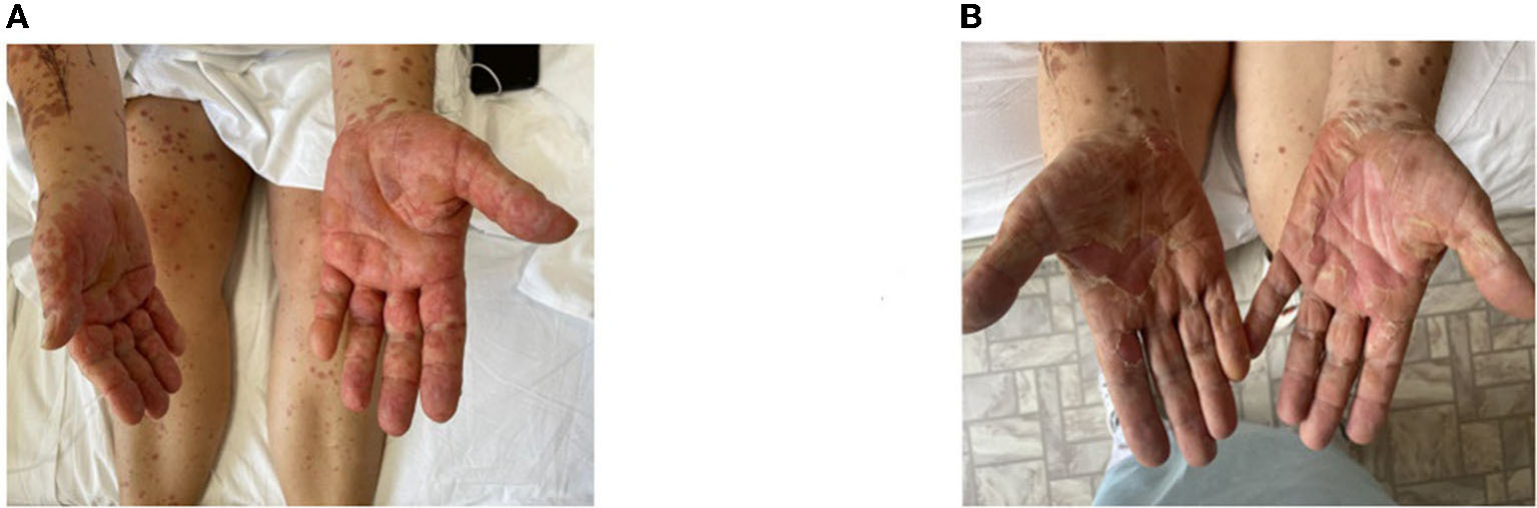
Skin process dynamics. **(A)** On the day of etanercept administration, the facial area with maculopapular rashes prone to confluence and hemorrhagic rashes were visible on the skin in the area of the red border of the lips. **(B)** On day 11, the facial area with foci of depigmentation and mucous membranes without signs of a pathological process were observed.

A moderate leukemoid reaction (leukocytosis up to a maximum of 11.9 × 10^*^9/l) was observed due to the high dose of sGCS. On day 10, the leukocyte level returned to the reference values without any further increase. Positive dynamics of the skin process were also observed (Figures 2–4). On day 5 after etanercept administration, complete epithelialization with foci of hyperpigmentation was observed.

The patient was discharged from the hospital on day 12 in satisfactory condition, and further follow-up was recommended. No etanercept-related adverse events were observed during the 6 months of follow-up.

## Discussion

Drugs from the NSAID group may have different risk levels for SJS/TEN development, including high-risk representatives: oxicams (piroxicam and meloxicam) (30). In our case, we suggest that etoricoxib may have been the causative agent. Although the absolute risk of developing SJS/TEN associated with the use of NSAIDs is low, it should be considered when monitoring patients who have recently started therapy. In three independent databases, the coxib group had a higher risk of developing SJS/TEN than other commonly used NSAIDs (31). Etoricoxib, a selective inhibitor of cyclooxygenase 2 (COX-2), is used in acute and chronic inflammatory diseases, such as osteoarthritis and gouty arthritis, and as a symptomatic therapy for pain syndromes (32). Several cases of SJS/TEN, multiform exudative erythema (33–35), even with fatal outcomes after etoricoxib have been described (36).

The pathogenesis of these syndromes is only partially understood. Cytotoxic T lymphocytes (CTLs), key cells in the pathogenesis of SJS/TEN, recognize the culprit drug, which is represented by HLA class I molecules on keratinocytes (37, 38). The literature provides data on several important pathways for the development of cell death, such as Fas-Fas-L-dependent, perforin/granzyme, and nitric oxide synthase-associated pathways (8, 39–44). The increase in pro-inflammatory mediators is enhanced by the cytotoxic effect, including the production of TNF-α, a potent pro-inflammatory driver. In TEN, the level of TNF-α expression in keratinocytes and macrophages is increased, as well as the perivascular expression of TNF-α in the damaged areas (42). It is noteworthy that bullae elements have a high concentration of TNF-α (22). I. Viard-Leveugle et al. showed that CTLs actively secrete a large amount of TNF-α (8).

The proposed involvement of TNF-α in the pathogenesis of SJS/TEN provides a new perspective. In a prospective, open-label, randomized study of etanercept vs. glucocorticosteroids in the treatment of SJS/TEN by Chuang-Wei Wang et al. etanercept was shown to reduce the predicted mortality based on SCORTEN (predicted vs. observed values of 17.7 vs. 8.3%, respectively) and to shorten the skin epithelialization time (mean epithelialization time was 14 vs. 19 days for etanercept and corticosteroids, respectively; *P* = 0.010) (22). Chuang-Wei Wang et al. showed a decrease in TNF-α and granulysin secretion in bulla-derived fluid in the plasma of patients with SJS/TEN treated with etanercept (45.7–62.5% reduction after treatment; all *P* < 0.05) and a parallel increase in Treg population (2-fold after treatment; *P* = 0.002) (22).

In the study by Paradisi et al. 10 patients with TEN were treated with TNF-a inhibitors. The authors reported that all patients had a rapid and complete response characterized by complete re-epithelialization without complications or side effects (95% CI for the observed mortality rate (i.e. 0) ranging from 0 to 30.8%). The healing time varied from 7 to 20 days (median: 8.5 days) (20). The same authors in another study described 17 patients who received a single injection of etanercept 50 mg. The patients were assessed according to SCORTEN, and the lowest score was 2, with seven patients having SCORTEN >5. Two patients died in the highest risk category (SCORTEN > 4). A total of 15 patients responded rapidly to treatment and achieved complete re-epithelialization (median time to healing: 8.5 days); no complications or adverse events were reported (21).

The clinical case we described was not treated with TNF-α inhibitors as monotherapy, but successful clinical cases of etanercept treatment as monotherapy have been reported in the literature (23–25). In 2022, Ao et al. published a cohort study of 25 patients with SJS/TEN that found that the combination of etanercept and sGCS significantly reduced the duration of the acute phase of the disease, shortened the hospital stay, and the time required for skin reepithelialization compared with the sGCS monotherapy group (26). Zhang J. et al. retrospectively analyzed 242 patients with SJS/TEN from Taiwan and China. Patients treated with the combination of etanercept and corticosteroids had a lower actual mortality rate than the corticosteroid monotherapy group (0 vs. 6.63%). There was a trend toward a predicted lower mortality rate with etanercept plus corticosteroids compared to sGCS monotherapy (95% CI: 0 [1.80–3.59], 0.71 [0.83–2.64], *P* = 0.006). The combination showed a reduction in skin healing time (median 12.0 days [8.5–14.0]) (13.0 [10.0–18.0]) (*P* = 0.004 and *P* = 0.012, respectively) and a lower incidence of gastrointestinal bleeding (*P* = 0.001) compared to sGCS monotherapy (27).

To sum up, the available results together with the presented case bring a new promising perspective for the use of TNF-α inhibitors in patients with SJS/TEN; however, most data sources are single case reports or case series, which limit the proof of the concept (8, 20–27). The current stage of data collection requires the accumulation of global clinical experience to answer open questions with reliable results. Larger cohort studies and meta-analyses are needed. International collaboration in data collection can be a key point to improve the understanding of the pathogenesis, treatment, and outcomes of this rare, life-threatening disease.

## Conclusion

Based on our clinical experience and the publications to date, the use of TNF-a inhibitors has pathogenetic validity and great potential in the treatment of SJS/TEN. In our opinion, etanercept therapy can be considered not only as one of the alternative therapies for SJS/TEN cases resistant to sGCS therapy but also as a first-line option for immunosuppressive monotherapy in patients with SJS/TEN. It is important to continue further studies to understand the time when TNF-a inhibitor therapy is most effective, as well as the phenotype of patients in whom this therapy would be most efficient.

## Data availability statement

The original contributions presented in the study are included in the article/supplementary material, further inquiries can be directed to the corresponding author.

## Ethics statement

Written informed consent was obtained from the participant/patient(s) for the publication of this case report.

## Author contributions

EN: literature review, literature collection and analysis, data analysis, and writing and editing the article. DF: literature review, literature collection and analysis, and writing and editing the article. UM, SA, YS, TK, and MSL: literature review, literature collection and analysis, and editing the article. AK and MAL: project supervision. All authors made substantial contributions to the conception of the work, the acquisition, analysis, interpretation of the data, the draft revision, the final approval of the version to be published, and agree to take full responsibility for all aspects of the work presented.
